# GABA Expression and Regulation by Sensory Experience in the Developing Visual System

**DOI:** 10.1371/journal.pone.0029086

**Published:** 2012-01-05

**Authors:** Loïs S. Miraucourt, Jorge Santos da Silva, Kasandra Burgos, Jianli Li, Hikari Abe, Edward S. Ruthazer, Hollis T. Cline

**Affiliations:** 1 Montreal Neurological Institute, McGill University, Montreal, Quebec, Canada; 2 Cold Spring Harbor Laboratory, Cold Spring Harbor, New York, United States of America; 3 Stony Brook School of Medicine, Stony Brook University, Stony Brook, New York, United States of America; 4 Departments of Cell Biology and Chemical Physiology, The Scripps Research Institute, La Jolla, California, United States of America; University of Southern California, United States of America

## Abstract

The developing retinotectal system of the *Xenopus laevis* tadpole is a model of choice for studying visual experience-dependent circuit maturation in the intact animal. The neurotransmitter gamma-aminobutyric acid (GABA) has been shown to play a critical role in the formation of sensory circuits in this preparation, however a comprehensive neuroanatomical study of GABAergic cell distribution in the developing tadpole has not been conducted. We report a detailed description of the spatial expression of GABA immunoreactivity in the *Xenopus laevis* tadpole brain at two key developmental stages: stage 40/42 around the onset of retinotectal innervation and stage 47 when the retinotectal circuit supports visually-guided behavior. During this period, GABAergic neurons within specific brain structures appeared to redistribute from clusters of neuronal somata to a sparser, more uniform distribution. Furthermore, we found that GABA levels were regulated by recent sensory experience. Both ELISA measurements of GABA concentration and quantitative analysis of GABA immunoreactivity in tissue sections from the optic tectum show that GABA increased in response to a 4 hr period of enhanced visual stimulation in stage 47 tadpoles. These observations reveal a remarkable degree of adaptability of GABAergic neurons in the developing brain, consistent with their key contributions to circuit development and function.

## Introduction

During the development of the central nervous system (CNS), synaptic strength and specificity mature together with, and influenced by, spontaneous and early sensory-evoked activity [1], [2], [3], [4]. In addition, synaptic release of γ-aminobutyric acid (GABA), which mediates fast synaptic inhibition in the mature nervous system [5], [6], [7], also plays multiple key roles as sensory circuits undergo functional development [8]. For instance, the mild disruption of GABAergic neurotransmission found in mice lacking the 65 KD isoform of the GABA-synthetic enzyme glutamate acid decarboxylase (GAD65) prevents these animals from entering the highly plastic critical period for ocular dominance plasticity in the visual cortex, a deficit that can be reversed by enhancing inhibitory transmission with benzodiazepines [9], [10].

*In vivo* data obtained in the developing retinotectal system of *Xenopus laevis* tadpoles, a important model system for the study of visual system development, indicate that GABAergic transmission is required to establish a functional balance between excitatory and inhibitory inputs which in turn contributes to activity-dependent maturation of receptive fields [11], [12], [13], [14]. Another important aspect of the *Xenopus laevis* tadpole model is that the developing CNS functions to process sensory information and motor activity necessary for the survival of the tadpole, even while extensive neurogenesis and circuit remodeling is occurring [15], [16], [17]. This situation creates a dual role for GABA in regulating both activity-dependent circuit maturation and in contributing to stable function of the existing network. Such developmental plasticity in CNS circuits suggests that the functional and anatomical circuits rearrange as neurons remodel and establish new sets of connections. Although the distribution and synaptic connectivity of GABAergic neurons in the tectum has been described in adult frogs [18], [19], little is known about the anatomical distribution of GABAergic neurons during tadpole development, or whether the neuroanatomical reorganization of GABAergic elements occurs during the period of circuit formation.

Numerous studies have demonstrated homeostatic regulation of GABAergic synaptic function in response to alterations in sensory input in vivo or neuronal activity in vitro [20], [21], [22]. Although homeostatic regulation of inhibitory function following sensory deprivation paradigms has been demonstrated during development [21], [23], the time-course and mechanisms by which enhanced sensory input affect GABAergic function in the developing brain are not yet clear.

In this study we examine the anatomical distribution of GABAergic neurons in the developing *Xenopus laevis* tadpole brain, and focus on changes in the visual system that occur as the circuit becomes functional. We found that the GABAergic cell distribution in the optic tectum reorganizes between stage 40–42 and stage 47 from an initially clustered to a sparse distribution of somata. Furthermore, we assayed the effects of brief periods of enhanced visual stimulation or brief visual deprivation on GABA levels in the optic tectum. Visual stimulation rapidly increased levels of GABA in the optic tectum, providing evidence for stimulus-evoked homeostatic regulation of inhibition in the developing retinotectal system.

## Materials and Methods

### Tadpole developmental staging

All animal protocols were approved by the Institutional Animal Care and Use Committees of Cold Spring Harbor Laboratory (protocol # 05-02-04) or the Montreal Neurological Institute (protocol #s 5801 and 5071). Albino *Xenopus laevis* embryos were isolated at neurulation, stage 23, and reared at 16°C in a 12 hrs dark/12 hrs light cycle until the selected developmental stage for analysis (staging according Nieuwkoop and Faber) [24].

### Tissue preparation

For anatomical experiments, tadpoles were anesthetized and fixed at the same time of day at the end of the dark cycle. Visual stimulation experiments started at the end of the dark cycle, and animals were sacrificed immediately after the visual stimulation protocol. These experiments were performed on tadpoles from several different clutches.

Animals at stages 40–42 and stage 47 were anesthetized in tricaine methanesulfonate (0.02% MS 222, Sigma, St. Louis, MO) in 0.1× Steinberg's solution or Modified Barth's Solution H and rapidly dissected to remove the skin and dura mater to expose the brain. For cryostat sections, tadpoles were transferred to freshly prepared 4% paraformaldehyde and 0.1% glutaraldehyde (Electron Microscopy Sciences, Fort Washington, PA) in phosphate buffer (PB, pH 7.4), exposed to a 15 sec microwave pulse and allowed to fix for 2 hr at room temperature. For eye immunostaining, whole animals were fixed intact by immersion in fixative. After two rinses in PB, specimens were cryoprotected overnight at 4°C in 30% sucrose (Sigma), after which they were transferred into Tissue-Tek O.C.T. Compound (PELCO International, Redding, CA) and cut into 20-µm horizontal, coronal, or sagittal sections. The combination of horizontal, coronal and sagittal planes of section were valuable for revealing the three-dimensional distribution of the GABA immunoreactivity. For L.R. White-embedded sections, the tissue was prepared as described by [25] with the following changes. The fixative used was 4% paraformaldehyde and 0.25% glutaraldehyde in 1 M sodium cacodylate buffer. The tadpole was exposed to an 8 second microwave pulse after which the tissue was left in fixative for 30 minutes at room temperature. After rinsing in PBS containing 3.5% sucrose, the tissue was quenched in 50 mM glycine in PBS and dehydrated in a graded series of ethanols (45 s each at 350 W in microwave). The tissue was infiltrated in L.R. White resin overnight at 4°C, embedded in gelatin capsules, and polymerized at 50°C. Floating sections were cut at 200 nm with an ultramicrotome using a Jumbo Histo Diamond Knife (Diatome). The sections were mounted on subbed glass slides (coated with 0.1% gelatin and 0.01% chromium potassium sulfate) and placed on a hot plate (∼60°C) for 10 minutes.

### Electron Microscopy

Electron microscopy studies were conducted as described in [26]. Briefly described, tadpoles at stage 47 were anesthetized as described above and fixed in a mixture of 2% paraformaldehyde, 2% glutaraldedyde, and 0.02% CaCl2 in 0.035 M sodium cacodylate buffer. The brain were postfixed in 2% osmium tetroxide; dehydrated in an acetone series; and infiltrated with Epon 812 resin. After sectioning the tectum at 70 nm, we used a rabbit polyclonal antibody against GABA (Sigma, St. Louis, MO) at a dilution of 1∶500 and a goat-anti rabbit IgG conjugated to 15 nm gold particles (Amersham, Arlington Heights, IL) to reveal the distribution of GABA [26].

### Immunostaining

All antibodies used in this study were obtained commercially. For all experiments performed in the present study, we performed control experiments in which the primary or secondary antibody was omitted. No labeling was obtained under these conditions. In addition, incubation of tissues with increasing dilutions of the primary antibody resulted in gradual diminishing and eventual disappearance of immunochemical staining.

To characterize the distribution of GABA-immunoreactive cells in the *Xenopus laevis* tadpole brain, background fluorescence for antibody labeling on cryostat sections was quenched with 50 mM ammonium chloride. Sections were permeabilized (1.0% Triton X-100; Sigma) and pre-incubated in blocking solution containing 5% normal goat serum (NGS; Gibco, Grand Island, NY) in 1% Triton X-100 for 1 hour, followed by incubation in a monoclonal rabbit anti-GABA primary antibody (Sigma; A0310) at 1∶1000 in 2% NGS in 0.1% Triton X-100 overnight at 4°C. After rinsing several times in PBS, sections were incubated for 2 hr in secondary antibody (Alexa Fluor 488, goat anti-mouse, Invitrogen, Eugene, OR) in PBS with 2% NGS and 0.1% Triton X-100. Slides were rinsed in PBS and coverslipped in Vectashield Mounting Medium with propidium iodide (PI; Vector Laboratories, Burlingame, CA) to counterstain nuclei.

For experiments on activity-dependent regulation of GABA immunoreactivity, sections were permeabilized (1.0% Triton X-100; Sigma) and pre-incubated in blocking solution containing 5% normal goat serum (NGS; Sigma) in 1% Triton X-100 for 1 hour followed by application of a monoclonal mouse anti-GABA primary antibody (Sigma; A0310, 1∶1000) and a polyclonal chicken anti-ßIII-tubulin antibody (Millipore, ab9354, 1∶500) in 2% NGS in 0.1% Triton X-100 overnight at 4°C. After rinsing several times in PBS, sections were incubated for 2 hr in secondary antibodies (AlexaFluor-488, goat anti-mouse and AlexaFluor-555, goat anti-chicken from Invitrogen, Eugene, OR) in PBS with 2% NGS and 0.1% Triton X-100. GABA and ßIII-tubulin immunostaining were always performed simultaneously. Slides were rinsed in PBS and coverslipped in Fluoromount G medium (Electron Microscopy Sciences, Hatfield, PA). Although the sensitivity of anti-ßIII-tubulin antibody labeling was compromised by the presence of glutaraldehyde in the fixative [17], it still serves to normalize the GABA immunoreactivity.

### Visual stimulation

For visual stimulation experiments, tadpoles were placed in a dark chamber (control) or a chamber with a 3×4 panel of green light-emitting diodes, with each row turning on and off sequentially (0.2 Hz cycle: 1 sec per row followed by 1 sec of darkness) for a period of 4 hr. This simulated motion stimulus has been previously described in detail [27]. Immediately after treatment, tadpoles were fixed and processed for GABA and ßIII-tubulin immunoreactivity as described above.

### Image acquisition

For anatomical analysis, sections were imaged using an Olympus BX50WI microscope with Olympus Fluoview FV300 confocal unit equipped with a LUMPlanFl/IR 60× water immersion objective (Olympus, 1.1 N.A.). To measure relative levels of GABA and ßIII-tubulin immunoreactivity, 20 µm horizontal sections were imaged on a Zeiss LSM 5 Pascal confocal mounted on a Zeiss Axioskop 2 FS microscope equipped with a Plan-Neofluor 25× (Carl Zeiss, 0.8 N.A.). Images of corresponding sections were acquired for each animal as 12-bit stacks of 20 focal planes (1 µm step). Identical settings were used for all sections for every animal. To minimize bleedthrough, confocal images of the AlexaFluor-488 and AlexaFluor-555 staining were obtained sequentially using an argon laser at 488 nm and a HeNe laser at 543 nm.

### Quantification of changes in GABA immunofluorescence

ImageJ software (National Institutes of Health, USA) was used for image analysis. For each channel we first subtracted background intensity, and performed a Z-projection summation of all 20 optical sections. Regions of interest (ROIs) were selected in the neuropil and cell body regions of the optic tectum and were analyzed independently. For every ROI, the mean intensities of GABA and ßIII-tubulin immunofluorescence were measured. For each animal this analysis was performed on 2 consecutive horizontal tissue sections, taken at 40 µm and 60 µm, below the dorsal surface of the brain. To quantify GABA immunofluorescence levels, each measurement was expressed as mean intensity value of the GABA signal normalized by the mean intensity value of the ßIII-tubulin signal to control for potential differences in tissue treatment between animals.

### ELISA to quantify GABA levels

A commercial enzyme-linked immunosorbent assay kit (GABA Research ELISA, Labor Diagnostika Nord, Nordhorn, Germany) was used to measure GABA levels in the midbrain of *Xenopus laevis* tadpoles. Immediately following the visual stimulation protocol, tadpoles (n = 8 for each group) were anaesthetized in solution containing 0.02% MS-222 and a GABA transaminase inhibitor, vigabatrin (100 µM; Sigma, V8261). Midbrains were quickly dissected out, frozen on dry ice and kept at −80 C until analysis. On the day of analysis, midbrains were homogenized in 0.01 N hydrochloric acid containing 1 mM ethylenediaminetrtraacetic acid (EDTA) and 4 mM sodium metabisulfite. Samples were divided into 4 aliquots and processed in parallel according to the manufacturer's instructions.

## Results

### Immunoreactivity profile of cells in the *Xenopus* visual system

**Figure 1:**
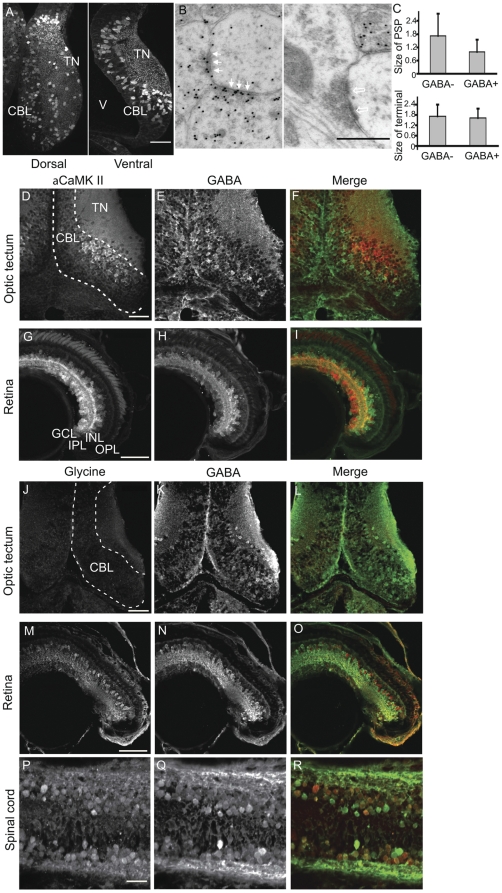
Immunocharacterization of stage 47 *Xenopus laevis* visual system. A. GABA immunofluorescent labeling in 200 nm LR White-embedded horizontal sections of optic tectum. GABA-positive somata are scattered throughout the cell body layer (CBL) and constitute the majority of neurons in the tectal neuropil (TN). The proliferative cells lining the ventricle (V) and in caudal tectum are not GABA immunoreactive. B. Ultrastructure of GABAergic synapses identified by post-embedding immunogold labeling in 70 nm sections from epoxy-resin embedded tissue. Electron micrograph of the tectal neuropil, showing two GABAergic presynaptic profiles forming symmetric contacts with a non-GABAergic postsynaptic profile (solid arrows). On the right a postsynaptic profile receives an asymmetric, non-GABAergic synaptic input with a prominent postsynaptic density (hollow arrows). C. Size comparison for GABA-negative (N = 28) and GABA-positive (N = 12) post-synaptic profiles (PSPs) and presynaptic terminals. D–F. Cryosections through optic tectum immunostained for αCaMKII (D) and GABA (E), and the merge of a CaMKII (red) and GABA (green) immunolabeling (F). There is little overlap of the CaMKII- and GABA-immunolabeled cells. G–I. αCaMKII (G) and GABA (H) immunolabeling in the retina. Most RGCs are αCaMKII immunoreactive. Neurons in the INL are predominately GABA-immunoreactive. Double labeling the retina for αCaMKII (H) and GABA (I) immunopositive cells shows little overlap in the cell body layers. J–L. Immunostaining for glycine and GABA reveals no detectable glycine label in the tectum (J). M–O. In the retina glycine-positive amacrine cells (M) are prominent in the INL (red in O) and are distinct from the GABAergic amacrine cells (N), shown as green in O. A few GABA-positive displaced amacrine cell bodies are also found in the ganglion cell layer (H, I, N, O). P–R. Immunolabeling for glycine (P, red in R) and GABA (Q, green in R) in the spinal cord shows neurons in the spinal cord can be immunoreactive for both transmitters (R). Scale Bars in A: 150 µm, in B: 500 nm, in D, G, J, M, and P: 50 µm, and apply to all images in the corresponding row.

The optic tectum is the primary sensory relay for visual inputs coming from the retina in fish and amphibia. As GABA has been identified as the principal inhibitory neurotransmitter in the optic tectum, we examined its distribution in the tectum of *Xenopus*. Figure 1A shows 200 nm horizontal LR White-embedded sections through the tectum of stage 47 tadpoles, which are labeled with GABA antibodies. The tectal neuropil, where axons of retinal ganglion cells and other tectal inputs contact tectal neuron dendrites, is located laterally in the tectum, and can be readily distinguished from the medial cell body layer, where most of the tectal somata are densely packed (Fig. 1A). The tectal neuropil is labeled in a punctuate pattern and GABA-immunoreactive cell bodies are sparsely distributed in the neuropil. GABA-positive cell bodies are distributed throughout the tectal cell body layer of the tectum. Notably, the majority of the cell bodies scattered within the tectal neuropil are GABA-immunoreactive (Fig. 1A). Electron micrographs through the tectal neuropil labeled by the post-embedding immunogold method for GABA (Fig. 1B) reveal that GABA-immunopositive profiles form symmetric synapses onto GABA-negative profiles. We also find GABA-positive presynaptic terminals synapsing on GABA-positive postsynaptic profiles (data not shown). GABA-positive (n = 12) and GABA-negative (n = 28) presynaptic terminals are similar in size. In contrast, the profiles postsynaptic to GABA immunoreactive terminals tend to be smaller than those postsynaptic to GABA-negative profiles (Fig. 1C).

The neurotransmitters, glutamate, GABA and glycine contribute to neuronal activity in the optic tectum at stages 46–48 [28]. Work in embryonic *Xenopus* tadpoles has suggested that the neurotransmitter identity of developing neurons may be more labile than previously believed, such that expression of neurotransmitter markers changed in response to different patterns of activity [29], [30]. To establish whether neurons in the optic tectum of stage 47 tadpoles expressed unique or multiple markers of neurotransmitter phenotype, we double-labeled sections of retina and tectum for GABA, glycine and the alpha isoform of the calcium/calmodulin-dependent protein kinase II (αCaMKII), a marker of mature excitatory neurons [31]. At stage 47, αCaMKII immunopositive neuronal somata are located close to the tectal neuropil (Fig. 1D). GABA-immunoreactive somata were distributed more broadly throughout the cell body layer (Fig. 1E, F). In the eye, the most dense distribution of αCaMKII immunopositive somata was found in the ganglion cell layer (GCL), with fewer labeled cells in the inner nuclear layer (INL) (Fig. 1G). Staining in the inner plexiform layer (IPL) was consistent with high levels of αCaMKII in the dendrites of retinal ganglion cells. We found that immunostaining for GABA and αCaMKII in neuronal somata in both optic tectum (Fig. 1D–F) and retina (Fig. 1G–I) was almost entirely non-overlapping, strongly suggesting that αCaMKII and GABA immunostaining label distinct populations of neurons. Interestingly, we found no glycine immunopositive somata in the optic tectum (Fig. 1J–L), however we did observe a faint punctate signal in the neuropil and in the interstices of the cell body layer, consistent with the possibility of glycinergic axons from cells outside the tectum, or of restricted glycine accumulation in terminals of glycinergic neurons. In the retina, we found glycine-immunopositive somata in the INL and IPL (Fig. 1M–O). In the IPL these are likely to be glycinergic amacrine cells and their arbors [32], [33]. Glycine and GABA-immunoreactivity were non-overlapping in somata in the INL (Fig. 1M–O). In the spinal cord we found numerous glycine immunopositive somata, however in contrast to the retina, many of these appeared to also be GABA-positive (Fig. 1P–R).

Taken together, these data show that GABA-immunoreactive cells in stage 47 *Xenopus* tadpole are broadly distributed across the entire optic tectum, and are distinct from αCaMKII-expressing and glycinergic cells. GABAergic cells in the tectum have no evident stratification or laminar distribution at this stage, although they are prevalent in the tectal neuopil. Finally GABA-immunoreactive tectal cells make synaptic contacts with both GABAergic and non-GABAergic profiles in the neuropil, forming the main source of inhibition in the optic tectum.

### Shift in distribution of GABA immunoreactivity across brain regions

**Figure 2:**
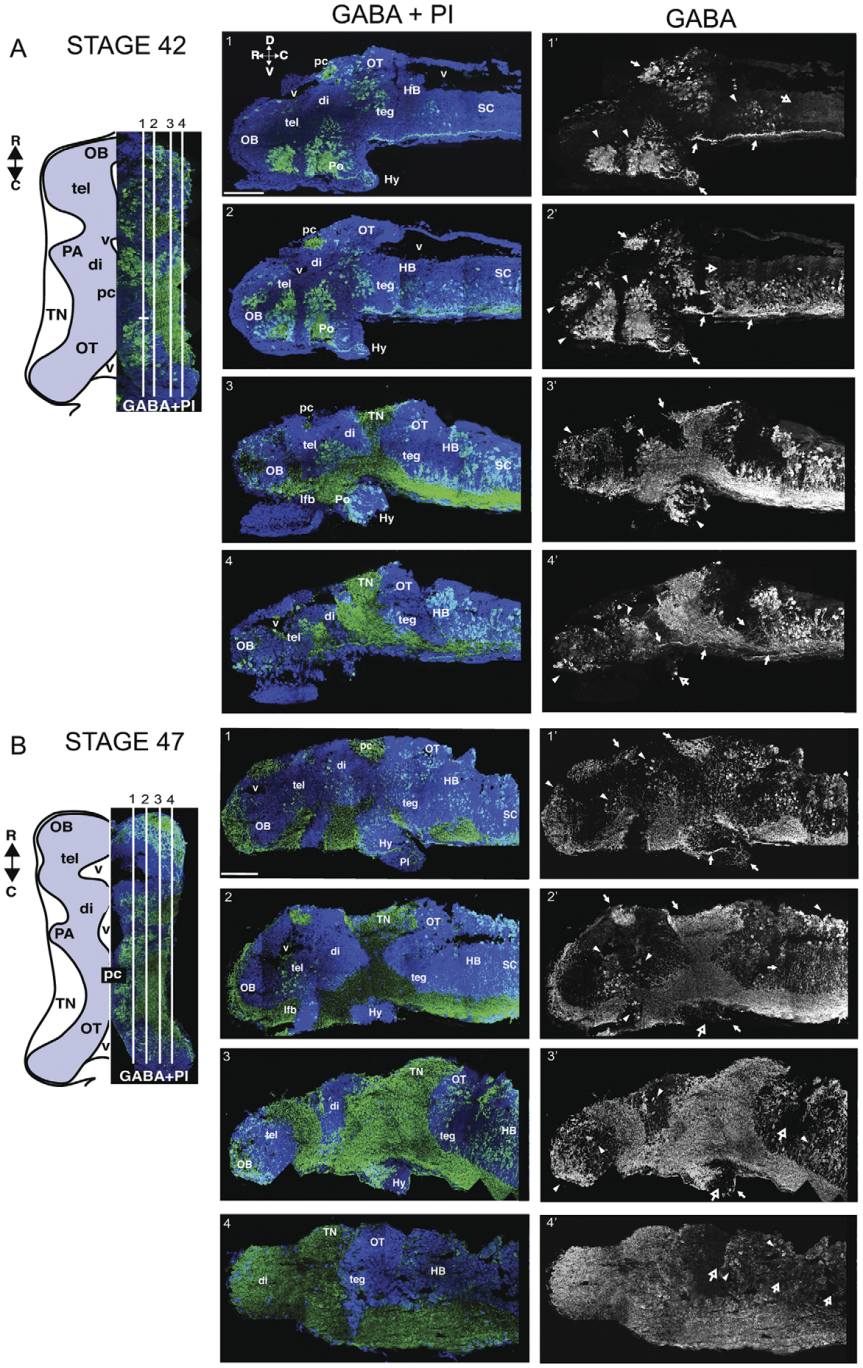
Distribution of GABA immunoreactivity in stage 42 and stage 47 tadpole CNS. A. Stage 42: Schematic (left) indicating relative positions of montaged sagittal sections of the tadpole brain. Blue is the cell body area; white is the neuropil area. Sections (1–4) show GABA immunostaining (green) counterstained with the nuclear label, propidium iodide (PI, blue). GABA staining alone is presented in the right panels (1′–4′). In panels 1′–4′ *arrowheads* indicate GABA containing somata, *filled arrows* are GABA-positive axon tracts, and *open arrows* denote GABA-sparse zones. B. Sagittal series through a stage 47 tadpole brain. The pattern of GABA-immunoreactivity in the brain is similar to stage 42 except for a dispersion of the dense clusters of GABA immunoreactive cells seen in younger brains and the vast expansion of the labeled cell body regions, neuropil and axon tracts in the older tadpoles. Scale bars, 250 µm. See text for details.

The redistribution of neurons from their birthplace to their final destination is an extremely important step in brain development. Between developmental stages 42 and 47, when tadpoles go from surviving on yolk to scavenging for food and avoiding predators, the *Xenopus* CNS undergoes significant structural and functional reorganization. During this time, synaptic transmission also matures dramatically [11], [34], [35]. We compared the GABA-immunoreactive labeling in stage 40/42 and stage 47 tadpoles. The pattern of GABA immunoreactivity in a representative stage 42 tadpole is shown in a series of sagittal sections in Fig. 2A. Medially, GABA immunoreactivity is present in two areas in the olfactory bulb (OB) which contain clusters of labeled cell bodies (Fig. 2A, sections 1, 2, arrowheads) and two spatially distinct populations in the ventral diencephalon (di) and the optic tectum (OT, Fig. 2A, sections 1–3). The hypothalamus (Hy) contains anteriorly situated GABA-immunoreactive cell bodies and positively stained projections at its periphery (Fig. 2A, sections 1–3). A high density of intensely GABA-immunoreactive neuronal processes was present in the posterior commissure (pc, Fig. 2A, sections 1–3; arrows), tectal neuropil (TN, Fig. 2A, sections 3, 4; arrows), and lateral forebrain bundle (lfb, Fig. 2A section 3). At the midbrain-hindbrain boundary, GABA-immunonegative proliferative cells in the caudal tectum abut a zone of GABA-positive somata in the rostral hindbrain (HB, Fig. 2A, sections 3 and 4). The dorsal fibers and the periventricular areas of the hindbrain are negative for GABA staining (Fig. 2A, sections 1, 2; hollow arrows). Long range GABA-immunoreactive axons are present in the ventral spinal cord (SC, sections 1–4, arrows).

Between stages 40/42 and 47, an important anatomical maturation of the brain has taken place that can be appreciated in the low magnification images of sagittal brain sections (Fig. 2B). The rapid proliferation of cells, as well as the expansion of fiber tracts and neuropil areas, contributes to a massive expansion of the volume of the brain and various brain regions. With this brain development, the olfactory bulb, midbrain, including the optic tectum and tegmentum (teg), and hindbrain show particularly large increases in GABA immunoreactive cell bodies, neuropil and projection fibers. As brain areas expand, the relative allocation of GABAergic neurons between different areas appears to remain relatively constant. On the other hand, the organization of GABA-immunoreactive neurons within each region takes on strikingly different distributions. In the hypothalamus, distinct GABA-immunoreactive somata and neuronal projection fibers are visible in the periphery of the hypothalamus (Fig. 2B, sections 1–3; arrows) as in the younger stages, but in contrast, in the older animals very few GABA-immunoreactive somata or projection fibers are located centrally (Fig. 2B, sections 2, 3). This exclusion of GABAergic somata in the hypothalamus appears to be an exception to the general developmental trend observed in nearly all other brain areas in which GABA-immunoreactive cells become more diffusely distributed with age. We examined this phenomenon in more detail using the developing midbrain as an example, where the reorganization of GABA-immunoreactive cells is particularly striking.

### Developmental reorganization of GABA-immunoreactive neurons in the optic tectum

**Figure 3:**
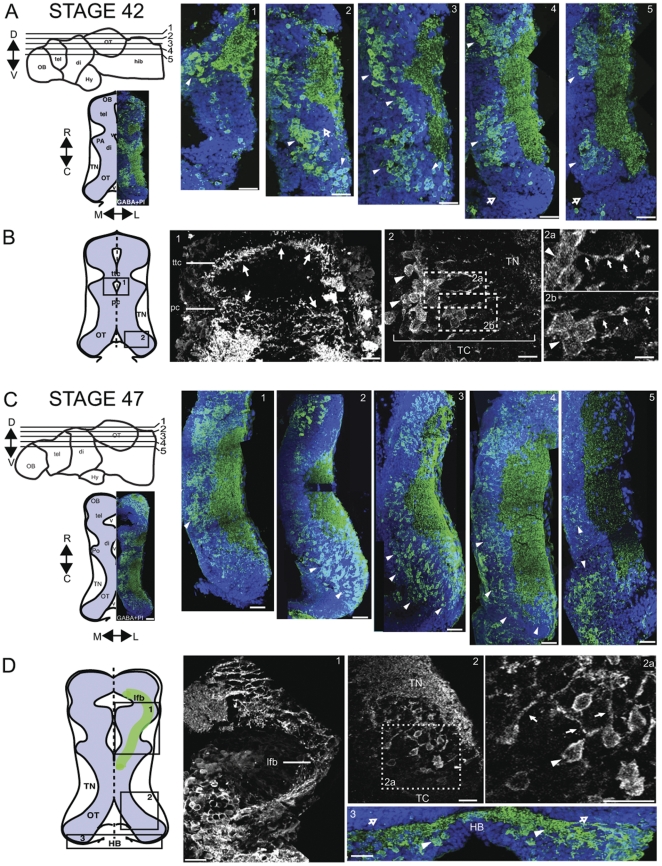
GABA immunoreactivity in optic tectum of stage 42 and stage 47 tadpoles (horizontal plane). A. Stage 42: Left: Schematic indicating relative positions of horizontal sections through the dorsal midbrain and locations of major brain regions (top left). Schematic of a horizontal section through the brain with locations of brain regions labeled. Blue is the cell body area; white is the neuropil area. An image of a GABA-immunolabeled right hemisection is superimposed on the schematic (bottom left). Sections (1–5) show GABA-immunoreactivity (green) counterstained with PI (blue). B. Schematic of horizontal brain section (left) showing regions of high magnification images, shown to the right. Higher magnification single optical sections from stage 42 midbrain. B1. Intense GABA immunolabeling of axons in the tecto-tegmental commissure (ttc) and posterior commissure (pc) (solid arrows). B2. Clustered GABA-immunoreactive neurons in the optic tectum (solid arrows) extend processes toward the neuropil. B2a, b. Enlargements of boxed regions in B2 showing GABA-immunoreactive processes (arrows) extending from labeled cell bodies (arrowheads in 2a,b). C. Stage 47: Left. Schematics comparable to A. Sections (1–5) of GABA-immunoreactivity (green) and PI counterstain (blue). GABA-immunoreactivity becomes more broadly distributed across the optic tectal cell body layer (arrowheads) and neuropil. D. Higher magnification (single optical sections) showing strong GABA labeling in the lateral forebrain bundle (lfb, D1), and sparse GABA-positive somata in the caudolateral optic tectum (D2a, arrowheads) extending GABA-positive processes toward the neuropil (D2a, solid arrows). D3. The border between the caudal optic tectum and the medial hindbrain (HB) shows that the proliferative zone in caudal tectum is negative for GABA immunostaining (arrows), whereas neuronal cell bodies and processes in the medial HB are GABA-immunolabeled (arrowheads). Scale bars, A, C: 50 µm; B1, 2: 20 µm; B2a,b: 10 µm: D1,3: 30 µm; D2a,b: 20 µm.

Patterns of GABA immunoreactivity in the midbrain at stage 40/42 and stage 47 are shown in a series of horizontal sections in Figure 3. Horizontal sections progressing from dorsal to ventral (sections labeled 1–5) through the right midbrain are shown in panels to the right of diagrams of the brain (Fig. 3A,C). Double-labeling with propidium iodide, to visualize cell nuclei, shows that GABA immunoreactivity is present in distinct clusters in the cell body regions and that Pl-labeled cell bodies that are not GABA-immunoreactive fill the cell body layer. (Fig. 3A). At stage 40/42, we find GABA-immunoreactive somata in clusters with relatively few GABA-immunoreactive cells scattered outside of these clusters, and robust GABA immunolabeling in the tectal neuropil (TN, Fig. 3A,B). A cluster of cell bodies is positioned rostrally and medially in the dorsal-most section and extends caudally and laterally through more ventral sections. GABA-immunoreactive cells are densely packed within the clusters (arrowheads). The proliferative zone at the caudal end of the tectum is devoid of GABA-immunoreactive cells (hollow arrows; Fig. 3A sections 4 and 5). Higher magnification optical sections of the tecto-tegmental commissure and posterior commissure reveal intense GABA immunoreactivity of long range projecting axons (Fig. 3B section 1). The clustered GABA-immunoreactive tectal cells (Fig. 3B section 2; arrowheads) extend processes with GABA-positive labeling toward the neuropil (Fig. 3B insets 2a, b; filled arrows).

In contrast, the sequence of horizontal cryosections of stage 47 animals (Fig. 3C), from dorsal to ventral, exemplifies the more scattered distribution of GABA-immunoreactive cell bodies within the optic tectum at this stage. Although there are some clustered GABA-immunoreactive cells close to the midline within the dorsal mesencephalon (Fig. 3C, section 1; arrowhead), in more ventral sections, GABA-positive somata do not appear to be arranged in tight clusters but rather are dispersed among GABA-negative cells throughout the cell body region (Fig. 3C, sections 2–5; arrowheads). In addition, by stage 47 scattered GABA-immunoreactive cells extend further caudally within the tectal cell body layer (Fig. 3C, sections 2–5), with caudolateral parts of the optic tectum containing a scattered population of tectal cells that are strongly GABA-immunoreactive (Fig. 3D, section 2). High magnification images of the tectum show GABA-immunoreactive cell bodies extending processes toward the neuropil (Fig. 3D, section 2a). At stage 47 the lateral forebrain bundle, which projects from the rostro-medial forebrain just caudal to the olfactory bulb into the midbrain (Fig. 3D left section) and serves as a diagnostic landmark [36], is strongly GABA immunoreactive. The border between the caudal optic tectum and the hindbrain is also a clear landmark, where GABA-immunoreactive neurons and processes in the hindbrain are apposed to GABA-negative proliferative cells in the caudal tectum (Fig. 3D, panel 3).

**Figure 4:**
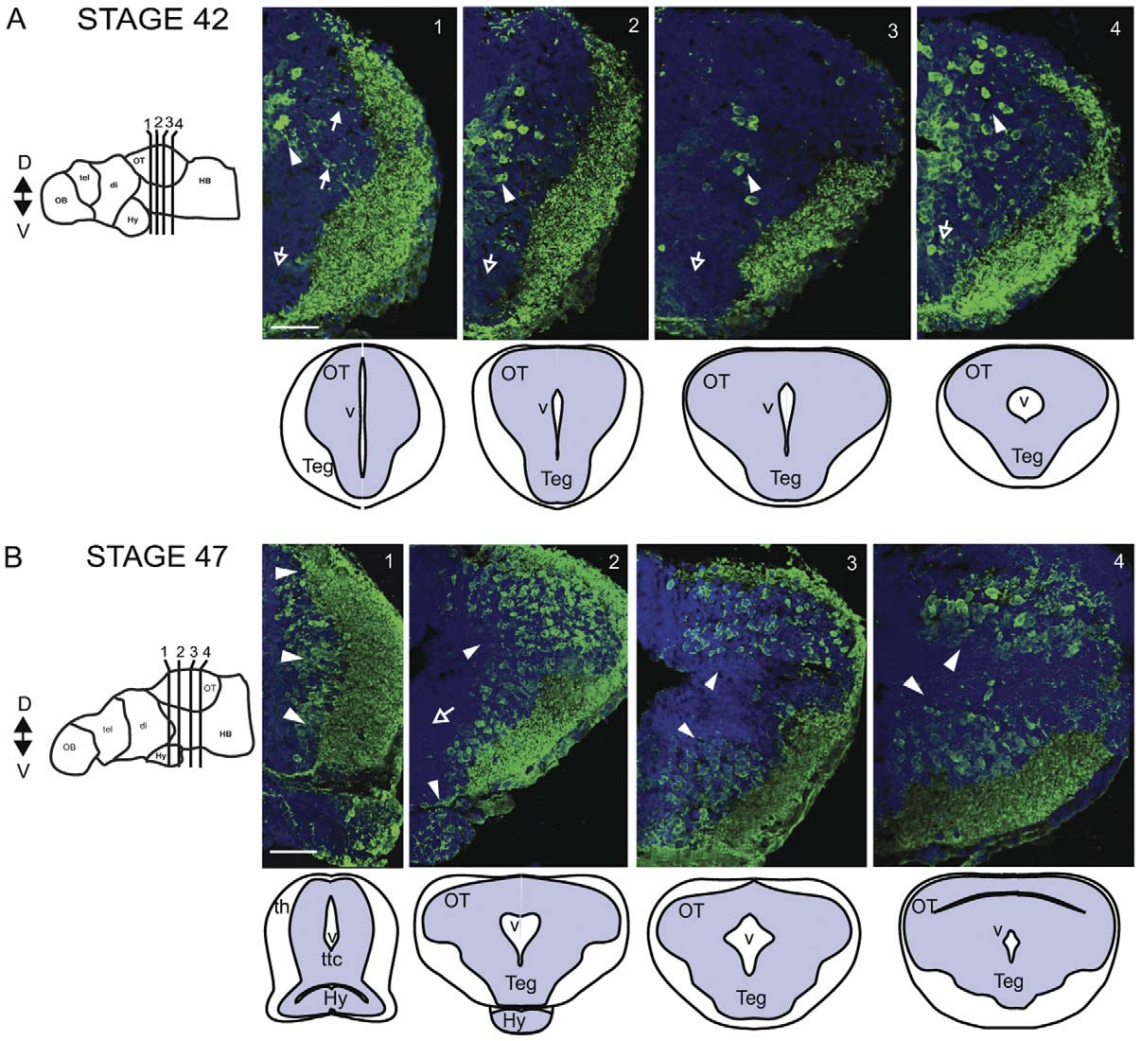
GABA immunoreactivity in optic tectum of stage 42 and stage 47 tadpoles (coronal plane). A. Stage 42: Left: Schematic indicating relative positions of coronal sections through the midbrain. Right: Sections (1–4) show GABA immunoreactivity (green) with PI counterstain (blue). Schematics under each section identify major brain regions in the sections. Blue is the cell body area; white is the neuropil area. GABA-positive cells are clustered medially in the anterior tectum (arrowheads) and send processes to the neuropil (solid arrows; section 1). A cluster of GABA-labeled neurons extends from the anterior ventricular region posteriorly and laterally within the tectum (sections 1–3, arrowheads). GABA-positive neurons are dispersed in caudal tectum (section 4, arrowhead). The tegmentum of stage 42 tadpoles has relatively few GABA-immunoreactive neurons (open arrows, sections 1–4), but extensive GABA-immunoreactivity in the lateral neuropil. B. Stage 47: Schematics shown are comparable to those in A. GABA-positive cells are interspersed throughout the optic tectum dorsally and in the tegmentum. The labeled neurons are distributed more laterally than in the younger tadpoles (arrowheads; sections 1–4). The zone closest to the tectal ventricle is largely devoid of GABA-immunoreactivity (section 2, hollow arrow). The tectal and tegmental neuropil regions are intensely GABA immunoreactive. Scale bar, 50 µm.

The anterior to posterior series of coronal sections through stage 40/42 and stage 47 optic tectum further elucidates the pattern of GABA-immunoreactive cell bodies and processes in the midbrain (Fig. 4). At stage 40/42 and stage 47 GABA-immunoreactive cells are clustered medially in the anterior tectum (arrowheads) and send processes to the superficial neuropil (solid arrows; Fig. 4A, section 1). Consistent with the pattern of GABA immunoreactivity seen in horizontal sections, the cluster of GABA-immunoreactive neurons in the anterior ventricular region (section 1) extends as a band posteriorly and laterally within the tectum (Fig. 4A, section 1–3 arrowheads). Coronal sections also reveal a population of GABA-immunoreactive cells that abut the ventricle in the caudal midbrain (Fig. 4A, section 4, solid arrow). The coronal sections clearly demonstrate the large increase in number and change in distribution of GABA immunoreactive neurons and processes in the optic tectum and tegmentum at stage 47. The anterior to posterior sequence of coronal sections further illustrates that GABA-positive cells are more interspersed throughout the optic tectum and the tegmentum in the stage 47 animal (Fig. 4B). In addition, in comparison to the coronal sections in the younger stages, the distribution of GABA-positive cells tends to extend further laterally at stage 47. Taken together, these observations show that a redistribution of neuronal somata, clearly evident in the optic tectum, takes place during development between stage 40/42 and stage 47.

### GABA-immunoreactive cell distribution in the retina at stage 42 and stage 47

**Figure 5:**
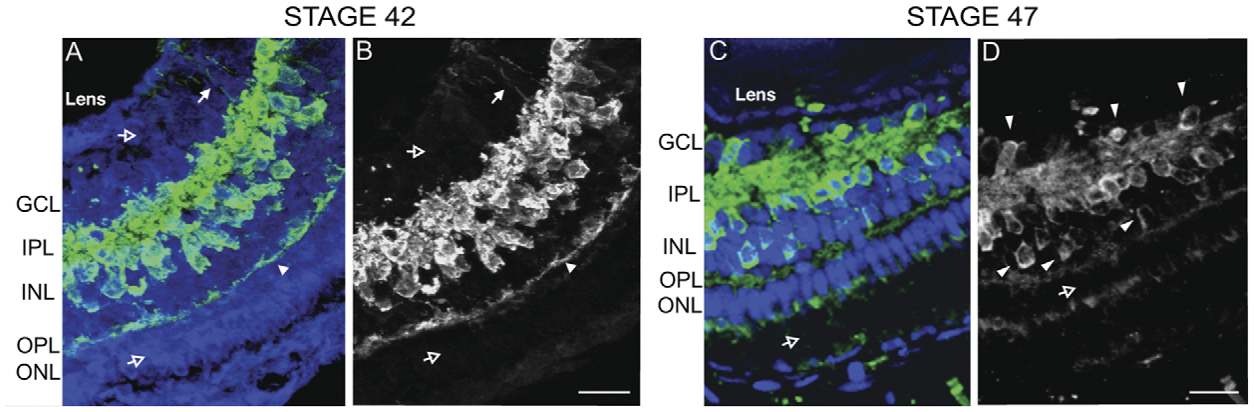
GABA immunoreactvity in retina of stage 42 and stage 47 tadpoles. A. Stage 42 coronal cryosection of the retina showing GABA immunoreactivity (green) and propidium iodide (blue) staining in the retina. B. GABA immunolabeling alone. At this stage, GABA immunolabeling is absent from the ganglion cell layer (GCL, hollow arrow) and outer nuclear label (ONL, hollow arrow). GABA-positive cell somata are densely packed in the INL and ramify processes into the GCL (solid arrow) and OPL (solid arrow). D. Stage 47 sections through retina showing GABA immunoreactivity (green) and propidium iodide counterstain (blue). A few GABA-positive somata are now evident in the GCL (arrowheads). GABA immunoreactivity is present in the IPL, INL and OPL, but absent in the ONL. Scale bar, 25 µm.

The retina is highly laminated. Retinal ganglion cells (RGCs) form the inner most cell body layer. The inner nuclear layer (INL) consists of amacrine cells, horizontal cells and bipolar cells. The outer nuclear layer consists of photoreceptors. Plexiform layers are the processes of the cells in neighboring nuclear layers. In stage 42 tadpoles, we observed abundant GABA-immunoreactive cell bodies in the INL of the retina (Fig. 5A,B,C). GABA-immunoreactive cells in the INL of the adult frog retina have been classified mainly as amacrine cells, though rare GABA-immunoreactive cells resembling bipolar cells have also been identified [37]. GABA-immunoreactive fibers are present in both the inner and outer plexiform layers (IPL, OPL). In the stage 42 retina, cell bodies in the ganglion cell layer (GCL) were all negative for GABA immunoreactivity (open arrow in GCL), although lightly GABA-immunoreactive fibers were visible (arrow in GCL; Fig. 5 A, B). At stage 47, the INL remained densely GABA-immunoreactive (Fig. 5 C, D), however some GABA-immunoreactive somata can now be seen in the GCL, which may be displaced amacrine cells [38].

### Modulation of GABA levels in the optic tectum by visual stimulation

**Figure 6:**
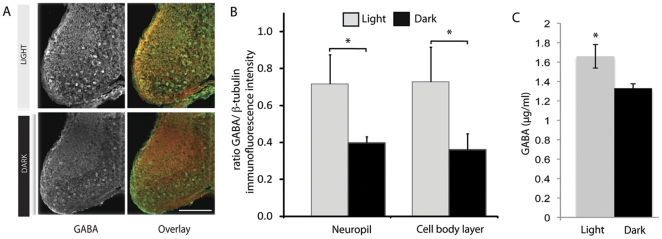
Modulation of GABA levels in the optic tectum by visual stimulation. A. Examples of cryosections from stage 47 midbrains immunostained for GABA and ßIII-tubulin. Tadpoles were either visually stimulated (n = 5) or kept in the dark (n = 4) for 4 hr. Scale bar, 100 µm. B. Animals exposed to visual stimulation had consistently higher levels of GABA immunoreactivity, normalized to ßIII tubulin, in both the neuropil and cell body layer compared to animals kept in the dark (*p<0.05, Student's t-test). C. Elisa measurements of GABA concentrations in homogenates of optic tectum are significantly higher in animals exposed to 4 hr of visual stimulation compared to animals kept in the dark.

Previous studies have shown that visual inputs activate GABAergic tectal neurons and that GABAergic inhibition plays an important role in visual information processing [11], [12], [13], [14], [39]. To test whether altered levels of visual input are able to modulate levels of GABA in the optic tectum, we exposed stage 47 animals to a simulated motion stimulus produced by an array of LEDs for 4 hr, as previously described [27]. This stimulus has previously been shown to drive the maturation of retinotectal excitatory transmission [40], increase dendritic arbor growth rates [27], and increase neuronal excitability and signal detection in the visual system [41]. Control animals were kept in the dark for 4 hr (see methods). We compared the amounts of GABA in the tectum by measuring the ratios of GABA and ßIII-tubulin immunofluorescence intensities in sections 40 µm and 60 µm below the dorsal surface of optic tectum (Fig. 6). We normalized levels of GABA immunofluorescence against ßIII-tubulin immunofluorescence to correct for any fluctuations in staining intensity that may have come from differences in postmortem treatment of the tissue. All immunostaining was performed on sections from matched sets of control and visually stimulated animals using the same solutions. For each section, regions of interest consisting of the tectal neuropil and the cell body layer were analyzed separately for immunofluorescence intensity. Significantly higher levels of GABA immunoreactivity (whether normalized against ßIII-tubulin or not) were observed both in the cell body layer and in the tectal neuropil in sections from animals that had been exposed to enhanced visual stimulation for 4 hr (N = 5) compared with animals kept in the dark (N = 4) for 4 hr (Fig. 6, two-tailed Student's t-test, p<0.05 Table 1).

**Table 1:** Quantification of immunofluorescence intensity for ßIII-tubulin, GABA and the GABA/ßIII-tubulin ratio, measured in tectal neuropil and the cell body layer from animals exposed to visual stimulus or dark.

	LIGHT			DARK		
Region	ßIIItubulin	GABA	ratio	ßIIItubulin	GABA	ratio
Neuropil	25.0±6.1	15.8±1.6	0.72±0.11	21.7±3.7	8.1±1.0	0.40±0.06
cell body layer	21.5±3.7	14.1±2.0	0.73±0.11	18.0±2.8	6.1±3.9	0.36±0.04

Fluorescence intensity values are in arbitrary units.

We also used ELISA to quantify GABA concentration in homogenates of optic tectum from tadpoles after 4 hours of enhanced visual stimulation or 4 hours of darkness. These measurements confirmed our finding of elevated GABA levels in visually stimulated animals (Fig. 6C, light = 1.66±0.21 µg/mL, dark = 1.34±0.08 µg/mL, 8 animals pooled per condition, and Table 2). Taken together, these results show that 4 hr of enhanced visual stimulation is sufficient to increase GABA levels in the optic tectum.

**Table 2:** Quantification of GABA levels (µg/ml) by ELISA.

aliquot	LIGHT	DARK
1	1.47	1.33
2	1.57	1.25
3	1.96	1.44
4	1.64	1.33
average	1.66±0.21	1.34±0.08

Tissue was collected from animals exposed to visual stimulation or kept in the dark. A tissue homogenate sample was divided into 4 aliquots and each aliquot was analyzed separately.

## Discussion

This study reports the anatomical distribution of GABA-immunoreactive neurons and processes in the developing *Xenopus laevis* tadpole brain. Interestingly, we find that GABAergic neurons are subject to significant reorganization during a period of development when they are actively participating both in sensory processing and plasticity. The positions of GABAergic neuronal somata undergo a systematic redistribution from clusters to a more uniform arrangement in the tadpole brain between stages 40/42 and stage 47. Moreover, we found that the levels of GABA in neurons of the optic tectum were rapidly increased by a brief period of enhanced visual stimulation, suggesting that activity-dependent regulation of the GABA synthetic enzyme GAD may play an important role in the homeostasis of circuit function.

GABA mediates most fast synaptic inhibition in the vertebrate brain and regulates network activity in the mature nervous system [5], [6], [7]. GABA also plays a pivotal role in circuit development, initially through its putative function as an excitatory neurotransmitter prior to the maturation of the hyperpolarizing chloride equilibrium potential in immature neurons [8], [11], [42] and subsequently by spatiotemporal regulation of neuronal activity as GABAergic neurons refine their synaptic connectivity [43]. Accordingly impaired GABAergic transmission has been linked to a number of developmental and neurological disorders such as epilepsy, schizophrenia, anxiety and drug abuse. Even as these diverse roles of GABAergic transmission have become increasingly well-established, the cellular mechanisms controlling the strength and efficacy of GABAerigic function are only starting to be clarified. To that end, the present study has examined the ontogeny and functional regulation of the GABAergic system during critical developmental stages in the functional maturation of a well-characterized brain circuit, the retinotectal system in *Xenopus laevis*.

### The retinotectal system of *Xenopus laevis* as a model for circuit development and function

The *Xenopus laevis* tadpole has proven to be an important experimental system for the study of vertebrate circuit development, owing to its amenability to live cell imaging, whole-cell electrophysiology, and targeted gene manipulation in the intact animal. In the developing *Xenopus* retinotectal circuit, GABAergic transmission regulates the timing and maturation of excitatory transmission and helps maintain a critical balance between excitation and inhibition [8], [11], [12], [13], [14], [44], [45]. Recent studies exploring the role of GABAergic neurotransmission in visual receptive field refinement in the *Xenopus laevis* tadpole optic tectum have established a requirement for well-regulated GABAergic transmission in this fundamental developmental process [12], [13], [14].

The widespread presence of GABA in the CNS of *Xenopus laevis* has been reported by immunohistochemical analysis [19], [46], [47], [48], [49] and by *in situ* hybridization of both of the GABA synthetic enzyme isoforms GAD65 and GAD67 [48]. We found populations of GABA-immunoreactive neurons in most regions of the brain, consistent with a widespread role of GABA in nervous system function [46]. GABA immunoreactivity labels neuronal somata, dendrites and axons and clearly reveal the presence of long projecting axons of GABAergic neurons, as has also been reported in other species [50], [51], [52], [53], [54], [55]. In both stages examined, GABA immunoreactivity was enriched in the telencephalon at the level of the olfactory bulb, in the preoptic region, optic tectum, hypothalamus, tegmentum and the spinal cord, consistent with observations in, among other species, leopard frogs [18], lamprey [52], [56], zebrafish and mouse [36]. In the retina, GABA-immunoreactive cells were seen in the retinal ganglion cell layer of stage 47 but not stage 40/42 tadpoles. These are most probably displaced amacrine cells, as described in the adult retina [57]. In addition, consistent with findings in other species, GABA staining was absent in regions that are known proliferative zones [36], [46], [47], [58]. Although GABA has been shown to have effects early in cell development and neuronal differentiation (see for review [59]), these data indicate that the source of GABA is from cells other than those in the proliferative zone.

### Clustered distribution of GABAergic neurons in the developing tadpole brain

In many instances, populations of GABAergic cells in the stage 40/42 animals occurred in spatially distinct clusters separated from one another by cells negative for GABA immunoreactivity. This was particularly prominent in the telencephalon (Fig. 2), but generally observed across the brain. This is a consistent finding across species [36], [47], and is thought to be related to regionalization of brain areas [36], [49], [60], [61], [62]. It has been proposed that the forebrain can be divided into six transverse domains, named prosomeres, defined by morphological or molecular criteria [63]. One characteristic of forebrain prosomeres is that progenitor mixing is prevented across boundaries [64], a contributing element in the subsequent emergence of distinct brain regions. This has led to the prosomeric model of forebrain development, a paradigm that emphasizes evolutionarily conserved topological regions and molecular expression associations in the neural tube. For example, expression of orthologs of the Distalless (Dll) family of homeodomain transcription factors correlates well with GABAergic neuron histogenic regions in numerous species. Indeed, in many forebrain regions of larval *Xenopus*, the expression pattern of GAD67 co-localizes with that of the Dll4 gene [48], however other regions rich in GABAergic cells do not express Dll genes, suggesting that there are other developmental regulatory genes involved in the determination of GABAergic cells in these regions [65]. This regionalization, considering the clustered distribution of GABAergic cells in young animals, may also reflect even more local subdivisions. The prosomeric model of development has become increasingly complex as more gene expression patterns continue to be identified and brain areas are further subdivided [66]. Perhaps the observation of densely packed clusters of GABAergic cells in stage 40/42 brains is due to their having been born and determined based on the expression of neurogenic and proneural genes that are spatially defined and confined to small regions [67]. These isolated clusters, if based on the combinatorial expression of genes that confer, or are involved with regional specification, could constitute clonal expansions of GABAergic subtypes. In this scenario the progenitors destined to generate GABAergic cells would expand in number and their differentiated progeny would remain in tight clusters as they begin to express their transmitter phenotype. Later in development, the regions may be invaded by non-GABAergic cells, resulting in the less densely organized distribution of GABA-immunoreactive cells in stage 47 tadpoles. While speculative, support for this idea can be drawn from distributive analysis of markers of interneuronal subtypes.

In the CNS, heterogeneous populations of neurons may be categorized based on their expression of various marker proteins. For example, distinct populations of inhibitory neurons can be distinguished by parvalbumin, calbindin and nitric oxide synthase (NOS) immunoreactivity [68], [69] and robust expression of αCaMKII is present in excitatory neurons [70], [71], [72], [73], [74]. In stage 47 *Xenopus* tadpoles, we found that strongly GABA and αCaMKII immunoreactive sub-populations were mutually non-overlapping in the optic tectum (Fig. 1D), suggesting that they may be ontogenetically distinct cell types. Similarly, in the retina the locations of GABA and αCaMKII immunoreactive somata were distinct (Fig. 1D), with more αCaMKII immunoreactive cells in the ganglion cell layer. In addition, in contrast to the spinal cord where glycinergic and GABAergic profiles are largely intermingled, in the retina these cells are segregated which might account for the distinct roles of glycinergic and GABAergic amacrine cell types in retinal function [75], [76], [77] Interestingly, we found no evidence for glycine-immunoreactive somata in the optic tectum, although a weak punctate distribution of glycinergic terminals was observed (Fig. 1E). This is in accord with electrophysiological recordings showing little or so contribution of glycinergic inhibition in the optic tectum of *Xenopus* tadpoles [11], [28]. Further histochemical or genetic [78] categorization of the diverse morphological classes of cells in the *Xenopus* optic tectum [79] remains to be systematically described.

### Homeostatic regulation of GABA levels in the visual system

Attenuation or hyperstimulation of neuronal circuit activity often leads to the activation of compensatory mechanisms that maintain the balance of excitation and inhibition within a functional range [20], [22], [80], [81], [82], [83]. In intact animals such compensatory mechanisms engaged in response to changes in sensory input have been shown to operate through homeostatic regulation of excitatory and inhibitory synaptic responses [23], [84], [85], [86], [87], [88] and neuronal excitability [41], and these mechanisms result in altered responses to sensory input [41], [89]. Mechanisms that contribute to homeostatic regulation of GABAergic inhibition in response to changes in network activity appear to include both changes in neurotranmission from GABAergic neurons and changes in neurotransmitter detection by postsynaptic neurons [20], [23], [90], [91], [92], [93]. Previous work in the adult primate visual cortex has shown that monocular deprivation decreases immunoreactivity for GABA and other proteins associated with GABAergic neurons in the ocular dominance bands corresponding to the deprived eye [71], [94]. Similarly, intraocular tetrodotoxin (TTX) and dark-rearing decrease GABA immunoreactivity and GABA_A_ receptor expression in adult animals [94], [95], [96]. In the adult rodent somatosensory system, whisker trimming leads to decreased GABA and GAD immunoreactivity in the corresponding barrels in the somatosensory cortex [97]. Visual deprivation in adult rats decreases the ratio of GABA receptors to AMPA receptors, and changes NMDA receptor properties, characteristic of more plastic juvenile cortex [92]. Homeostatic control of inhibitory transmission may be regulated differently during development [23]. During critical periods of sensory system development, when the numbers and strength of GABAergic synaptic contacts increase significantly [9], [98], [99], [100], dark rearing prevents the normal increase in GABAergic function [88], [99], [100]. Careful quantitative studies demonstrate that the number of GAD65 immunoreactive perisomatic puncta in layer 2/3 of visual cortex decreases in response to visual deprivation from birth and that subsequent visual experience increases the number in GAD65 immunoreactive boutons [101]. In the rodent somatosensory system, unilateral vibrissa removal in pups decreases the numbers of GABA-immunoreactive neurons in contralateral layer 4, but also resulted in changes in inhibitory circuitry in both ipsilateral and contralateral cortex [102]. Similar to studies in visual cortex, whisker removal from birth decreased the number of GABA immunoreactive synaptic inputs in layer 4, determined by electron microscopy [21], [103]. Our study, which focused on rapid responses to increased sensory input, revealed that just 4 hr of enhanced visual stimulation was sufficient to elevate GABA levels in the developing *Xenopus* optic tectum. This finding is consistent with a mechanism for rapid homeostatic regulation of GAD activity that that could function to constrain neuronal activity in the optic tectum, and maintain stable function over a wide range of circuit excitation levels during a developmental period when afferent and intrinsic activity may be experiencing substantial fluctuations as the maturing circuit undergoes dynamic structural remodeling and synaptic plasticity. The finding is also consistent with a role for sensory input in promoting the maturation of neurons and neuronal circuits. At this point, the mechanistic and functional interactions between experience-dependent maturation of circuits and experience-dependent homeostatic regulation of circuit function during development remain to be resolved.

Two isoforms of GAD are expressed in neurons. The GAD67 isoform is distributed throughout the neuron, is constitutively active, and accounts for 90% of GABA synthesis in neurons [104]. By contrast, GAD65 enzymatic activity is regulated by neuronal activity and is located selectively in nerve terminals [105], [106], [107], where it may be optimized to respond to activity-dependent cues to enhance GABA synthesis and vesicular packaging [108], [109], [110]. Based on the visual-stimulation mediated increase in GABA immunoreactivity in both the cell body region and neuropil of the optic tectum, it appears that the increase in GABA that we detect could arise from increased activity of both GAD67 and GAD65, consistent with changes in both somatic and presynaptic GABA immunoreactivity reported in other systems [21], [94], [95], [96]. In the early developing *Xenopus* embryo, blockade of signaling by early non-synaptic GABA or glutamate release has been shown to impact transmitter fate in spinal neurons, favoring an increased number of neurons subsequently expressing excitatory transmitters over neurons expressing inhibitory transmitters [29], [30]. These spontaneous activity-sensitive modifications of neuronal transmitter fate are restricted to a very early embryonic period, prior to functional synapse formation and are therefore quite different from the homeostatic regulation we observed in older tadpoles when tectal synapses are both functional and highly plastic.

Numerous studies have investigated the cellular mechanisms of homeostatic changes in inhibitory transmission. Chronic activity blockade in cultured cortical neurons decreases the strength of inhibition, detected as a decrease in the average amplitude of miniature inhibitory postsynaptic currents (mIPSCs), by decreasing postsynaptic GABAergic receptors [93]. This may be mediated by glial release of Tumor Necrosis Factor-α, which causes internalization of GABA receptors [111]. On the other hand, intense afferent stimulation in hippocampal area CA1 increased the strength of inhibitory connections by increasing GABAergic mIPSC amplitudes through what appeared to be an enhancement of presynaptic GABA content [91]. Another means by which the neurons can modulate the efficacy of GABAergic transmission is through control of their intracellular chloride concentration and the chloride driving force [112]. By contrast both visual deprivation and somatosensory can also lead to potentiation of specific inhibitory connections in the cortical circuit [88], [102], suggesting that homeostatic regulation of circuit function can be expressed as specific changes in strength of different types of connections within the circuit.

Retrograde Brain-Derived Neurotrophic Factor (BDNF) signaling through Tropomycin-Related Kinase receptor type B (trkB) and subsequent kinase activity at the GABAergic presynaptic site has been strongly implicated in the developmental and homeostatic regulation of GABAergic transmission in both developing and mature systems. *In vitro* studies in organotypic and acute brain slices have demonstrated up-regulation of GABAergic inhibitory synaptic number and strength, as well as increased levels of GAD65 in response to local BDNF release [113], [114] whereas a single-cell genetic knockout of BDNF causes a local reduction in inhibitory input [115]. Such rapid, acute control of GABAergic transmission likely operates in part through regulation of GAD65 activity. On a longer time scale, BDNF-TrkB signaling may directly regulate GAD65 expression levels through the activity-regulated CREB-dependent GAD65 transcription [116]. Similarly, the activity-regulated transcription factor, Npas4, appears to regulate a program of gene expression that controls the development of inhibitory connectivity [83]. Although BDNF has been shown to affect several aspects of retinotectal development [117], [118], [119], [120], [121], a particular role of BDNF in the development of GABAergic circuitry in the tectum has not yet been reported.
